# Professional autonomy in dealing with complications: discourse of obstetric nurses working in planned home births

**DOI:** 10.1590/0034-7167-2022-0388

**Published:** 2023-03-17

**Authors:** Natália Webler, Lilian Conceição Guimarães de Almeida, Jordana Brock Carneiro, Luana Moura Campos, Tanila Amorim Glaeser, Telmara Menezes Couto, Samara Garcia de Lima, Ingrid Bonfim Silva

**Affiliations:** I Universidade Federal da Bahia. Salvador, Bahia, Brasil; II SobreParto - Coletivo de Assistência ao Parto de Salvador. Salvador, Bahia, Brasil.

**Keywords:** Professional Autonomy, Nurses Midwives, Home Childbirth, Emergencies, Nursing., Autonomie professionnelle, Enfermeras Obstetrices, Parto Domiciliario, Urgencias Médicas, Enfermería., Autonomia Profissional, Enfermeiras Obstétricas, Parto Domiciliar, Emergências, Enfermagem.

## Abstract

**Objective:**

to understand the exercise of professional autonomy by obstetric nurses in dealing with complications in planned home births.

**Methods:**

this is a qualitative study, supported by the Discourse of the Collective Subject methodological framework, whose data collection took place from January to March 2021, through interviews guided by a semi-structured script, in which seven midwives who are members of a childbirth care collective in northeastern Brazil and who work in the home context participated.

**Results:**

four central ideas emerged from the collective discourses related to the exercise of professional autonomy by obstetric nurses: shared decisions; theoretical-practical instrumentalization; professional expertise; and teamwork.

**Final considerations:**

obstetric nurses’ autonomy in the face of complications reflects the safety of planned home births and is based on collective critical thinking, reinforcing this professional’ leading role in obstetrics.

## RESUMO

**Objetivo:**

compreender o exercício da autonomia profissional de enfermeiras obstétricas na condução de intercorrências em parto domiciliar planejado.

**Métodos:**

trata-se de um estudo qualitativo, amparado no referencial metodológico do Discurso do Sujeito Coletivo, cuja coleta de dados se deu no período de janeiro a março de 2021, por meio de entrevistas guiadas por roteiro semiestruturado, das quais participaram sete enfermeiras obstétricas integrantes em um coletivo de assistência ao parto do Nordeste do Brasil e que atuam no contexto domiciliar.

**Resultados:**

emergiram dos discursos coletivos quatro ideias centrais relacionadas ao exercício da autonomia profissional de enfermeiras obstétricas: decisões compartilhadas; instrumentalização teórico-prática; *expertise* profissional; e trabalho em equipe.

**Considerações finais:**

a autonomia das enfermeiras obstétricas diante das intercorrências reflete a segurança do parto domiciliar planejado e está pautada no pensamento crítico coletivo, reforçando o protagonismo desta profissional na atuação em obstetrícia.

## RESUMEN

**Objetivo:**

comprender el ejercicio de la autonomía profesional de las matronas en el manejo de las complicaciones en los partos domiciliarios planificados.

**Métodos:**

se trata de un estudio cualitativo, sustentado en el marco metodológico del Discurso del Sujeto Colectivo, cuya recolección de datos ocurrió de enero a marzo de 2021, a través de entrevistas guiadas por un guión semiestructurado, en el que participaron siete parteras integrantes de un colectivo de atención al parto en el Nordeste de Brasil y que actúan en el contexto domiciliario.

**Resultados:**

surgieron cuatro ideas centrales de los discursos colectivos relacionados con el ejercicio de la autonomía profesional de las matronas: decisiones compartidas; instrumentalización teórico-práctica; experiencia profesional; y trabajo en equipo

**Consideraciones finales:**

la autonomía de las enfermeras obstétricas frente a las complicaciones refleja la seguridad de los partos domiciliarios planificados y se basa en el pensamiento crítico colectivo, reforzando el papel de este profesional en obstetricia.

## INTRODUCTION

The consolidation of nursing, as well as obstetric nurses’ work (ON) was influenced by patriarchy, capitalism and, concomitantly, the hospital and medical-centric logic, which impacted the constitution of professional autonomy. In this sense, driven by the desire to change the contemporary obstetric context and the existing professional organization in it, ON identify in the context of planned home birth (PHB) a favorable space for their autonomous performance, which requires preparation, mainly, to act in the face of complications.

Since the propulsion of capitalism in the mid-16th century, the functioning of hospitals has followed a productivist logic. Likewise, nursing work, when institutionalized, was impacted by this system^(1-2)^. The functions performed by nurses in this space were manual in nature, attributed to domestic chores, following the industrial trend of the time of low wages and long working hours^(3)^. Added to this is the little social recognition of this work was also linked to the patriarchal structure that prevailed at the time, reinforcing the subservience of female work (figure of the nurse - woman) in the face of male domination (figure of the physician - man)^(4)^.

Similarly, the context of obstetric care was marked by the transition from the home birth scenario to the hospital environment, which aimed, following capitalist ideals, to increase the number of deliveries attended daily to accelerate productivity^(5-7)^. In this context, to this day, the pathologization movement of childbirth in the social imaginary is strengthened, which comes to be understood as a situation in which women are fragile and in suffering, demanding, therefore, assistance from a physician and their subordinate team to shorten the time of this event through interventions and safeguard mothers’ and fetuses’ lives^(8-10)^. Hence, control mechanisms are structured over women’s bodies, reverberating in the impairment of female autonomy and self-confidence and hostility of traditional midwives and other women involved in childbirth^(8-11)^.

This reality is perpetuated to this day, directing obstetric care to a hospital-centered model based on medical hegemony. In the Brazilian scenario, specifically, ON, in most institutions, including maternity hospitals, they have limited autonomy, mainly because they are subordinated to a medical team^(12-14)^. This reality is opposed to what is evidenced in the scientific literature and the World Health Organization guidelines, which point to the best maternal-fetal experiences of deliveries assisted by ON, associated mainly with a smaller number of interventions and implementation of good practices^(15-20)^.

Despite the dissemination of these studies, as well as campaigns and strategies, nationally and internationally, who recognize the benefits of ON’s work in the childbirth scenario and encourage it, these professionals still have not managed to reach space for their autonomous work in most public and private hospitals in Brazil^(12-14)^. These guidelines are also addressed in movements for the humanization of childbirth and birth, being the assistance of obstetric nursing a possible strategy for the dehospitalization and demedicalization of these physiological events^(21-22)^. Thus, there is a growing trend of PHB in Brazil, corroborating the current model in developed countries, a scenario in which ON has been consolidating itself as qualified professionals supported by its councils^(23-25)^.

In this way, the PHB scenario is configured as an expansion of ON’s area of operation. However, although it is a safe option for pregnant women at usual risk, maternal and fetal complications can occur during childbirth and birth, which requires specific knowledge and practical expertise to act. Thus, it is extremely important to understand how the autonomous exercise of obstetric nursing takes place in the face of these adverse situations, which demand decision-making and assertive conduct.

## OBJECTIVE

To understand the exercise of ON’s professional autonomy in dealing with complications in PHB.

## METHODS

### Ethical aspects

The present study was approved by the Research Ethics Committee of the *Universidade Federal da Bahia* Nursing School, in compliance with Resolution 466/2012 of the Brazilian National Health Council. It should be noted that, prior to the beginning of data collection, the responsible researchers, together with participants, read the Informed Consent Form.

It is important to inform that, at this moment, all participants were informed about the research objectives as well as the risks and benefits of participating in it. Moreover, they were also informed about speech recording and subsequent audio transcription with total preservation of identity anonymity. For data confidentiality, the interviewees’ names were replaced by a code composed of acronym ON, which represents obstetric nurse, followed by a random Arabic numeral, such as ON1, ON2. Aware of this information, professionals signed the document.

### Study design

This is a descriptive study with qualitative nature. For its elaboration, the researchers used the assumptions elaborated by Lefèvre and Lefévre, which compose the methodology entitled Discourse of the Collective Subject (DCS), anchored in the Social Representation Theory^(26)^. The use of these theoretical-methodological references makes it possible to achieve the social representation of a theme, preserving the individual sphere in a collective discourse^(26-27)^.

It is noteworthy that compliance with the checklist of COnsolidated criteria for REporting Qualitative research (COREQ) was a priority for the researchers throughout the course of research, considering its importance for the organizational excellence of work, linked to intervention reliability and material quality.

### Study setting

The study scenario was a childbirth care collective that provides assistance to PHB throughout the metropolitan region and is located in a capital in the Brazilian Northeast, this being a group of physicians and ON who call themselves a “collective” in view of work philosophy on which they base their practice, focused on principles such as transdisciplinarity and horizontality.

### Data source

The researchers intentionally elected ON as participants who met the pre-established inclusion criteria, such as being a member of a childbirth collective, working in PHB care and having experienced complications in this scenario. It should be noted that no participant fits the exclusion criteria, such as absence from work in the collective or no-show, for three consecutive times, to the scheduled interview.

### Data collection, organization, and analysis

Initially, the researchers, via telephone contact, invited participants, having obtained acceptance from seven ON, which made up the group of interviewees. After this stage, from November 2019, the researchers began to approach the collective through participation as listeners of the weekly scientific meetings and also of collective prenatal spaces they carried out. With this, it was possible to know the work developed by the collective of midwives (physicians and ON) and to establish a bond with them, which favored the security for their participation in the study.

In-depth individual interviews were carried out during January and March 2021 using Google Meet. It is worth mentioning that the COVID-19 pandemic, which marks the period, requires social distancing and, therefore, the virtualization of interviews. These moments were conducted by two graduate students, members of the research team who had previous experience in carrying out qualitative research. To carry out these moments, the researchers used a previously structured script as support, containing objective questions, regarding professional characterization, and subjective questions, to investigate the research object, which were guided by the following question: how is the exercise of your professional autonomy in handling complications in the scenario of PHB?

The interviews, whose average duration was 40 minutes, had their content transcribed in full by undergraduate nursing students involved in the project and previously trained for this activity. Interviewees’ speeches were transcribed, and their content validated by the researchers responsible for conducting the investigation, and then sent so that participants could also attest to its veracity.

For data organization and categorization, the DCS methodological framework was used. According to assumptions elaborated by Lefèvre and Lefèvre, the opinions expressed by participants relate to socially elaborated knowledge, bringing in its core a representation of what is thought by a collective^(26)^. Seeking to reveal this reality, the researchers systematized the testimonies by grouping individual statements that brought in essence the same key expressions. This step allowed the researchers to organize the speeches initially and appropriate their content, which then led to the identification of the meanings translated homogeneously by key expressions, thus reaching the main idea synthesis (MIS) expressed in its content and regrouping the excerpts from the interviews by similarity and/or complementarity^(26-27)^.

From the fulfillment of the aforementioned operations, considering ON as collective subjects, since they are inserted in a social group, it is possible to affirm that interview categorization allowed elaborating DCS, written in the first person singular as well as identifying the social representations brought by them^(27)^. In this way, these speeches function as a social mirror, since they contain meanings, senses and positions of society about a theme^(26-28)^, that, in the case of this study, is about the exercise of ON’s autonomy in the handling of complications in the PHB scenario. Subsequently, this information was interpreted and discussed based on current scientific literature.

## RESULTS

The study participants were seven ON, aged between 25 and 40 years, whose average is 32 years and mostly self-declared brown/black. As for training in midwifery, five of the interviewees completed specialization and two residency. Of the total, six ONs are linked to childbirth care institutions, such as maternity and birth centers. It is noteworthy that one of the professionals mentioned never having worked in the institutional model, which has been working in obstetrics for ten years, which took place entirely in the scenario of PHB. Regarding the average time of insertion of these professionals in the collective, it is noteworthy that this was in the range of three years, close to the average time of training in obstetrics, which is five years, pointing out that, in general, professionals started to integrate it soon after or shortly after finishing their graduate studies.

From the collective discourses, ON reveal that the exercise of professional autonomy permeates through shared decision-making, theoretical-practical instrumentalization, professional expertise and teamwork, as expressed in MIS that follow:

### MIS 1 - Shared decisions

The collective discourse revealed that the constructions of knowledge and agreements signed during prenatal care with women and others involved in the PHB scenario contribute to the exercise of ON’s autonomy in the process of assistance to complications. Clarified about the risks, a necessary co-responsibility is established for shared decision-making.

*During prenatal care, from the first meeting, we build a bond and work on co-responsibility with the woman and whoever is going to be at the birth. We explain about the most frequent complications and the possibilities of acting in front of each one. If during assistance one of these situations occurs, women find it easier to understand what is happening and make a decision together with the team. We also send them articles, protocols and consent forms and discuss them during consultations. All of this reinforces co-responsibility and makes us more confident to act. We know that complications can get worse, but the security that gives us autonomy and that reassures pregnant women is in the scientific knowledge we share and in the team’s practical experience.* (DCS1 - ON1, ON2, ON4, ON5, ON7)

### MIS 2 - Theoretical-practical instrumentation

The exercise of professional autonomy, as pointed out by ON, goes through training and constant theoretical-practical updating, based on current scientific evidence, which equip them to act in the face of complications. Access to this evidence is given by participating in courses, events, continuing education actions of the collective they are part of as well as searching for scientific material and sharing experiences with other professionals who work in PHB.

*We use what we have of scientific evidence, we don’t have a work plastered by institutional protocols. The most important thing is to be able to identify and reverse situations in a timely manner to have a positive outcome, whether stabilizing and resolving them at home or transferring. We seek continuous improvement and updating. In the case discussions that we held within the collective, we were able to understand the views of different professional classes about the assistance we provide and, with that, we added knowledge. We participate in theoretical and practical courses in obstetric and neonatal emergencies, which cover different topics such as postpartum hemorrhage, cord prolapse, resuscitation and other situations that may arise with women or newborns. To bring more quality to the assistance, we go to events that address innovations and current scientific studies, where we also exchange experiences with professionals who work in PHB in other places.* (DCS2 - ON1, ON2, ON3, ON4, ON6)

### MIS 3 - Professional expertise

ON also reveal, in their speeches, that the previous practical experience, acquired in the years of professional performance, provides security for the exercise of their autonomy in the face of complications in the PHB scenario. Such experiences sharpen the look of professionals in the face of what is beyond the physiological, which allows them to anticipate adverse situations.

*First, I learned that security lies in the autonomy of not intervening in the physiological course of childbirth. The fact that I have been working with home birth assistance for years allows me to have a quick perception when something is going beyond the physiological. There are situations and risk factors that make me alert and, therefore, I can anticipate complications. Working at a birth center, I was able to come into contact with numerous situations that require quick action by the team, which is formed, mostly by obstetric nurses, and I take the lessons learned from these experiences to home births. I consider it essential to master protocols and conducts as well as having all the material resources necessary for assistance and knowing how to use them. In order to feel safe in making decisions at a PHB, I think it is important to have a prior approximation with this reality, being supervised by the team, initially.* (DCS2 - ON2, ON3, ON4, ON5, ON6, ON7)

### MIS 4 - Teamwork

The collectivity expressed in the speech denotes that the synchrony between conducts during assistance to complications gives professional security, essential for the full exercise of autonomy. This happens through the alignment of behaviors, usually carried out in systematic team discussions, and horizontality, one of the principles of assistance in PHB.

*The other midwife who is in PHB needs to be aligned with me, have ownership over what is happening and dominate the work process in the face of an complication, because this gives us security to exercise autonomy. We work a lot with non-verbal communication. During a maternal complication, I perceived our synchronized work as a dance, in which we knew the steps to follow: one prepared the medication, the other installed the serum, then raised the legs, while I did uterine massage. Synchronous work is fully proportionate to assistance safety and saves time. In our work, one gives strength to the other and, together, we exercise our autonomy. When we are at home, even in a multidisciplinary team, there is no hierarchy, but a horizontality, different from what we see in institutions. Discussions of cases of complications in weekly team meetings are opportunities for us to work on horizontality and reinforce the autonomy of professionals.* (DCS4 - ON1, ON3, ON4, ON6, ON7)

## DISCUSSION

The collective discourse points out that the exercise of ON’s autonomy during handling of complications in the PHB scenario is linked to the sharing of decisions with women. This happens based on the construction of a bond between parties, which facilitates the establishment of a relationship of trust, contributing to dialogues about the possibilities of action in the face of conditions that deviate from expectations, even when there is a need for transfer^(28)^. In this sense, with everyone aware of the risks and benefits of each conduct, a co-responsibility is built between the team and the family for the decisions taken and their outcomes.

To build this co-responsibility, the PHB team needs to accept subjectivity issues that involve fears and insecurities of pregnant women and their families about childbirth and birth processes, helping them to empower them to make informed decisions^(29-31)^. Regarding the sharing of information during prenatal care, it is essential to mention that it must be done freely, distancing itself from a biased and/or imposing character, to focus, in fact, on illuminating ideas, possibilities and improving critical thinking, thus contributing to informed decision-making by pregnant women, which may even protect them from experiencing expressions of obstetric violence^(29-30)^. Thus, there is the essentiality of prenatal consultations, spaces in which the figure of the nurse has been pointed out as a facilitator of this process^(30-32)^.

In this follow-up, pregnant women are guided on the importance of reading quality scientific studies and also protocols and terms of consent, which helps to provoke a discussion in the consultations, thus favoring the understanding and mastery of the entire process^(33)^. Thus, a relationship of trust can be established between those involved, since the team is aligned with the reality of that family. The family is aware of the behaviors that may be taken by professionals during the assistance, which creates a favorable space for the exercise of autonomy by ON^(30,32-34)^. Considering that the most adverse situations present an emergency context, in which there is no time for consideration, the relevance of constructions and the establishment of agreements during prenatal care is even more important.

Dialoguing about complications with women in an informed manner enables professionals to individualize assistance, which occurs through care assessment and planning that adapts to each family’s particularities^(32-35)^. In the PHB scenario, this is facilitated since the dimensioning of human resources takes place in a 2:1 model, i.e., two midwives for a binomial, and the follow-up of these professionals extends from prenatal to puerperium^(35-37)^. It is worth noting that in the Unified Health System (*Sistema Único de Saúde*) context there are obstacles to the individualization of this care, what can be overcome with birth plan implementation, which can be built with the pregnant woman during prenatal care consultations in Primary Health Care and respecting the possibilities of maternity hospitals^(38-39)^.

It is important to emphasize that this is only possible considering the team’s theoretical-practical preparation in learning spaces, being essential to master the recommended obstetric procedures and knowledge of the available scientific evidence^(13,34-36)^. This instrumentation, associated with the possibility of acting without rigid protocols, unlike what happens in the hospital context, are factors that favor professional autonomy in the management of complications in PHB and directly impact the quality of care provided^(40-41)^. This is because the service provided by professionals is directly related, among other things, to their satisfaction and personal well-being, that results from a comfortable work environment to the realization of their professional autonomy as well as with the mastery of their work process^(12,14,40)^.

It is noted, however, that there are no established official protocols for assistance to complications at home, which demands that professionals develop protocols that, in general, are adapted from institutional assistance and may vary from teams’ specificities^(41-43)^. It is also noteworthy that the necessary adaptations in the protocols may permeate the lack of a formal referral and counter-referral system, especially linked to the Unified Health System, for adverse situations that require hospital transfers so that operational plans for these cases, previously established in the home birth planning, may undergo modifications in different locations in the country^(44-45)^.

In view of this reality, ON recognize the importance of constantly updating and expanding their theoretical knowledge, especially through participation in events, theoretical-practical courses and team case discussions as well as through the act of sharing experiences with professionals who carry out PHB in other places^(41-42,46)^. Added to this is the possibility of broadening the view of care based on case discussions, as it adds the different points of view of each professional class that makes up the collective.

The limitation of access to information about the possibility of PHB during graduation and, above all, in professional training processes in the obstetric area, which is a reflection of cultural prejudice with the theme, weaken the recognition of professionals regarding this possibility of assistance^(41-42)^. Faced with this reality, it is recommended that professionals who wish to work in this care model, in addition to adding up-to-date theoretical knowledge and professional experience in obstetric care, also seek with teams that are already active and experienced in the experience of childbirth care in this scenario^(36,40-42)^. In this regard, in the Brazilian scenario there is a pioneering work, carried out in the city of Recife, Pernambuco, entitled “Urban Parting Project: Training in Home Birth Care” (*Projeto Parteria Urbana: Capacitação em Atenção ao Parto Domiciliar*), theoretical-practical workload, whose objective is to train ON, midwives, among other professionals to monitor pregnant women and their families at PHB supported by scientific evidence and assumptions of humanization^(47)^.

The skills and abilities necessary for this action and, concomitantly, for the full exercise of autonomy, are associated with the ability to detect in a timely manner situations that are beyond the physiological, anticipating the possibility of complication, and acting quickly in favor of a quick and effective resolution, both in order to solve it at home and to perform the stabilization for a transfer. This reaffirms the maxim that mastery over their work process is directly associated with the full exercise of ON’s autonomy^(12-13,16,40-41)^. In this area, it is also essential to have material resources to reverse situations that arise, such as surgical instruments, medications, resuscitation kit, and, above all, recognizing the need and the ideal time for their use as well as knowing how to handle them properly^(25,46)^.

This does not mean, however, as signaled in the collective discourse, that there is a need to intervene in the physiology of childbirth so that professional autonomy to intervene when necessary cannot be confused with an abuse of power and, thus, trigger a cascade of invasive behaviors, resulting in a negative outcome of this experience for women and their children^(48)^. In the reality of professionals with previous experience in hospital institutions, where unnecessary interventions are routine, it may be necessary to deconstruct care practices^(41-42,46)^. In this regard, professionals who work in hospitals point to PHB as a space for deepening the understanding of physiology of childbirth, with the potential to transform the perception of the event and, above all, the way of acting in the obstetric scenario^(49)^.

It should be noted that the dynamics of work at PHB is also different with regard to the prevalence of horizontality among team members as well as prior alignment of behaviors to be adopted. This characteristic adds team well-being and satisfaction, favoring motivation for professionals in their work environment^(40)^ and, therefore, implies positive outcomes in home care. In this regard, some permanent education strategies that provide this dialogue and collective construction of knowledge should be prioritized^(50)^, as the case discussions in the team, as currently occurs in the aforementioned childbirth collective.

In this work perspective, it becomes possible to achieve an exercise of professional autonomy in the community, including in a multidisciplinary team, since this assistance distances nurses’ work from subordination and favors synchrony in professionals’ actions^(12-14,35)^ during assistance to complications. This joint work sometimes eliminates verbal communication, making decision-making and thus solving the problem more agile. A study reveals that nonverbal communication between human beings is as important as spoken language, because body expressions play an essential and significant role in the communication process^(51)^. In obstetrics, this type of interaction also occurs between ON and parturient women, fundamental for achieving harmony^(52)^ and dealing with decision-making processes.

It is noteworthy that teamwork favors the exercise of professional autonomy, since it adds knowledge and that professionals know that they can count on emotional and theoretical support from other colleagues to conduct care^(40,53)^, including complications. Thus, it is possible to state that this collective action is configured as a differential in the PHB scenario, since it is from the interaction between the professionals that security is added to decision-making and leading role and full autonomy of those who are in charge of this assistance in these circumstances are reinforced: ON.

### Study limitations

Considering that PHB assistance may vary according to the region of Brazil, especially since there are no institutionalized federal action protocols, the study is limited to transpose results emerging from the experiences facing the complications reported by ON of a single PHB collective.

### Contributions to nursing, health, and public policies

The research brings contributions to obstetrics and nursing by reinforcing the importance of ON’s autonomy in their different areas of action, considering their capabilities and skills, especially in the face of complications such as postpartum hemorrhage, cord prolapse and resuscitation. Furthermore, it draws attention to the indispensability of spaces for the theoretical-practical preparation of these professionals to work in the home scenario, including during graduate school. It should be noted that, in this area, it is also essential to incorporate theoretical guidelines on this subject during graduation, in order to raise awareness in students about the physiological character of childbirth and birth processes, with the positive experience in these events being directly related to professionals’ autonomy and, above all, of women and families who experience them, in a process of co-responsibility for decisions to be made.

Added to this, the research is also innovative in presenting the viability of PHB to obstetrics, pointing out that acting in this scenario constitutes a counter-hegemonic change movement, which requires greater disclosure about its potential. It is also noteworthy that, from the point of view of public health, the findings of this study consolidate the model of care advocated in public policy documents prepared by the Ministry of Health, since it is based on scientific evidence and is aligned with the principles of humanization of childbirth and birth.

## FINAL CONSIDERATIONS

The study highlighted that ON exercise their autonomy from the moment they share decision-making with the woman and family in the face of complications. This process, which takes place from the construction of a bond and theoretically based dialogues, makes the woman acquire confidence about the PHB model and the team, condition sine qua non for a relationship of co-responsibility to be established and, thus, the childbirth collective also feels safe to act in family assistance. This relationship between women and the team is supported by ON’s incessant search for current scientific evidence as well as their practical improvement, which gives them expertise to act in the face of complications. It should also be noted that the autonomy of these professionals is also a reflection of the partnership between them and the feeling of belonging to a community.

Considering the above, speeches denote the expansion of ON’s professional activities, which comes from the traditional category of midwives, who for many years exercised their autonomy at home in Brazil, but who over time distanced themselves from this due to the hegemonic medicalocentric logic. Thus, the study advances by bringing up the resumption of this place of collective assistance, but with a greater degree of autonomy, including with regard to the management of complications in PHB, which directly influences the quality of care provided to women and newborns.

## References

[1] Melo CMM. (1986). Divisão Social do Trabalho e Enfermagem.

[2] Dutra HS. (2016). Divisão social do trabalho e enfermagem. Rev Enferm UFPE.

[3] Melo CMM, Carvalho CA, Silva LA, Leal JAL, Santos TA, Santos HS. (2016). Nurse workforce in state services with direct management: revealing precarization. Esc Anna Nery.

[4] Cunha YFF, Sousa RR. (2016). Gênero e enfermagem: um ensaio sobre a inserção do homem no exercício da enfermagem. Rev Adm Hosp Inov Saúde.

[5] Davis-Floyd R. (2001). The technocratic, humanistic, and holistic paradigms of childbirth. Int J Gynaecol Obstet [Internet].

[6] Pimentel C, Rodrigues L, Müller E, Portella M. (2014). Autonomia, risco e sexualidade: a humanização do parto como possibilidade de redefinições descoloniais acerca da noção de sujeito. Rev Estud AntiUtilitaristas e Pós-Coloniais [Internet].

[7] Cheyney M, Davis-Floyd R. (2019). Birth in Eight Cultures.

[8] Ehrenreich B, English D. (2010). Witches, Midwives & Nurses: a history of women healers.

[9] Vasconcelos PP, Andrade BBF, Araújo KEAS, Medeiros HHA, Costa MSO, Correia MB (2020). Social media as a source of knowledge for the process of normal delivery. Cogitare Enferm.

[10] Pereira FM (2020). Parteiras, Medicina e Ciência: políticas do parto e diálogos necessários na atenção à saúde da mulher.

[11] Reis TLR, Padoin SMM, Toebe TFP, Paula CC, Quadros JS. (2017). Women’s autonomy in the process of labour and childbirth: integrative literature review. Rev Gaúcha Enferm.

[12] Saad DEA, Riesco MLG. (2018). Autonomia profissional da enfermeira obstétrica. Rev Paul Enferm [Internet].

[13] Costa RLM, Costa ILS. (2017). Um ponto de resistência: enfermagem, medicina e gênero no contexto hospitalar. Cad Esp Fem.

[14] Santos FAPS, Enders BC, Brito RS, Farias PHS, Teixeira GA, Dantas DNA (2019). Autonomy for obstetric nurse on low-risk childbirth care. Rev Bras Saude Mater Infant.

[15] Leal MS, Moreira RCR, Barros KCC, Servo MLS, Bispo TCF. (2021). Humanization practices in the parturitive course from the perspective of puerperae and nurse-midwives. Rev Bras Enferm.

[16] Duarte MR, Alves VH, Rodrigues DP, Souza KV, Pereira AV, Pimentel MM. (2019). Care technologies in obstetric nursing: contribution for the delivery and birth. Cogitare Enferm.

[17] Alves TTM, Paixão GPN, Fraga CDS, Lírio JGS, Oliveira FA. (2018). Role of the obstetric nurse in the development of labor and delivery. Rev Enferm Atenção Saúde.

[18] Silva LAT, Fonseca VM, Oliveira MIC, Silva KS, REG, Gama SGN (2020). Professional who attended childbirth and breastfeeding in the first hour of life. Rev Bras Enferm.

[19] World Health Organization (WHO) (2018). WHO recommendations: Intrapartum care for a positive childbirth experience [Internet].

[20] Organização Pan-Americana da Saúde (OPAS) (2018). Organização Mundial da Saúde (OMS). OMS define 2020 como ano internacional dos profissionais de enfermagem e obstetrícia [Internet].

[21] Lima PVSF, Soares ML (2015). Fróes GDR, Machado JR, Santos SM, Alves ED. Liga de humanização do parto e nascimento da Universidade de Brasília: relato de experiência. Rev Gestão Saúde.

[22] Conselho Federal de Enfermagem (Cofen) (2019). VII Marcha pela Humanização do Parto reforça importância de informação [Internet].

[23] Cursino TP, Benincasa M. (2020). Parto domiciliar planejado no Brasil: uma revisão sistemática nacional. Ciênc Saúde Colet.

[24] Denipote AGM, Lacerda MR, Nascimento JD, Tonin L. (2020). Parto Domiciliar Planejado no Brasil: onde estamos e para onde vamos?. Res, Soc Dev.

[25] Conselho Regional de Enfermagem (Coren-SC) (2016). Parto Domiciliar Planejado [Internet].

[26] Lefevre F, Lefevre AMC. (2005). O discurso do sujeito coletivo: um novo enfoque em pesquisa qualitativa.

[27] Lefevre F, Lefevre AMC. (2014). [Discourse of the collective subject: social representations and communication interventions]. Texto Contexto Enferm.

[28] Fox D, Sheehan A, Homer C. (2018). Birthplace in Australia: processes and interactions during the intrapartum transfer of women from planned homebirth to hospital. Midwifery.

[29] Venâncio DL. (2021). Comunicar em saúde com foco na humanização do atendimento à gestante e puérpera. Rev Pensar Acad.

[30] Kottwitz F, Gouveia HG, Gonçalves AC. (2018). Route of birth delivery preferred by mothers and their motivations. Esc Anna Nery.

[31] Gomes CBA, Dias RS, Silva WGB, Pacheco MAB, Sousa FGM, Loyola CMD. (2019). Prenatal nursing consultation: narratives of pregnant women and nurses. Texto Contexto Enferm.

[32] Dias BR, Oliveira VAC. (2019). Pregnant women perception on nursing care during habitual risk prenatal. Rev Enferm Cent-Oeste Min.

[33] Rabelo ARM, Silva KL. (2016). Care of the self and power relations: female nurses taking care of other women. Rev Bras Enferm.

[34] Clemons JH, Gilkison A, Mharapara TL, Dixon L, McAra-Couper J. (2021). Midwifery Job Autonomy in New Zealand: I do it all the time. Women Birth.

[35] Oliveira TR, Barbosa AF, Alves VH, Rodrigues DP, Dulfe PAM, Maciel VL. (2020). Assistance to planned home childbirth: professional trajectory and specificities of the obstetric nurse care. Texto Contexto Enferm.

[36] Committee on Obstetric Practice (2017). Committee Opinion No. 697: Planned Home Birth. Obstet Gynecol.

[37] Collaço VS, Santos EKA, Souza KV, Alves HV, Zampieri MF, Gregório VRP. (2016). Give birth and be born in new times: care provided in the puerperium by the Hanami Team. REME Rev Min Enferm.

[38] Gomes RPC, Silva RS, Oliveira DCC, Manzo BF, Guimarães GL, Souza KV. (2017). Delivery plan in conversation circles: women’s choices. REME Rev Min Enferm.

[39] Silva ALNV, Neves AB, Sgarbi AKG, Souza RA. (2017). Plano de parto: ferramenta para o empoderamento de mulheres durante a assistência de enfermagem. Rev Enferm UFSM.

[40] Bonfada MS, Moura LN, Soares SGA, Pinno C, Camponogara S. (2018). Autonomia do enfermeiro no ambiente hospitalar. Enferm Brasil [Internet].

[41] Santos LM, Mata JAL, Vaccari A, Sanfelice CFO. (2021). Trajectories of obstetric nurses in the care of planned home childbirth: oral history. Rev Gaúcha Enferm.

[42] Mattos DV, Vandenberghe L, Martins CA. (2014). Motivation of midwives for household childbirth plans. Rev Enferm UFPE.

[43] Koettker JG, Bruggemann OM, Freitas PF, Riesco MLG, Costa R. (2018). Obstetric practices in planned home births assisted in Brazil. Rev Esc Enferm USP.

[44] Valinho PB, Zveiter M, Mouta RJO, Seibert SL, Marques SC. (2021). The difficulties of planned home birth in Brazil: a systematic review. RSD.

[45] Pascoto GS, Tanaka EZ, Fernandes LCR, Shimo AKK, Sanfelice CFO. (2020). Difficulties in home birth care from the perspective of obstetric nurses. Rev Baiana Enferm.

[46] Mattos DV, Vandenberghe L, Martins CA (2016). The obstetric nurse in a planned household birth. Rev Enferm UFPE.

[47] Conselho Regional de Enfermagem (Coren-SP) (2012). Projeto multidisciplinar em Recife (PE) capacita profissionais de saúde em atenção ao parto domiciliar [Internet].

[48] Palharini LA. (2017). Autonomia para quem? o discurso médico hegemônico sobre a violência obstétrica no Brasil. Cad Pagu.

[49] Coddington R, Catling C, Homer C. (2020). Seeing birth in a new light: the transformational effect of exposure to homebirth for hospital-based midwives. Midwifery.

[50] Lima F, Martins CA, Mattos DV, Martins KA. (2018). Permanent health education as a strengthening of obstetric nursing. Rev Enferm UFPE.

[51] Padua LBO, Reinert AJ. (2020). A importância da comunicação não verbal para uma liderança organizacional eficiente. Rev Científ Multidisc Núcleo Conhecimento.

[52] Oliveira PS, Couto TM, Gomes NP, Campos LM, Lima KTRS, Barral FE. (2019). Best practices in the delivery process: conceptions from nurse midwives. Rev Bras Enferm.

[53] Amorim T, Araújo ACM, Guimarães EMP, Diniz SCF, Gandra HM, Cândido MCRM. (2019). Perception of obstetrical nurses on the care model and practice in a philanthropic maternity hospital. Rev Enferm UFSM.

